# Two complete mitochondrial genomes from *Praticolella mexicana* Perez, 2011 (Polygyridae) and gene order evolution in Helicoidea (Mollusca, Gastropoda)

**DOI:** 10.3897/zookeys.626.9633

**Published:** 2016-10-25

**Authors:** Russell L. Minton, Marco A. Martinez Cruz, Mark L. Farman, Kathryn E. Perez

**Affiliations:** 1 School of Science and Computer Engineering, University of Houston Clear Lake, 2700 Bay Area Boulevard MC 39, Houston, Texas 77058 USA; 2 Department of Biology, University of Texas Rio Grande Valley, 1201 West University Drive, Edinburg, Texas 78539 USA; 3 UK Healthcare Genomics, 225 Plant Science Building, 1405 Veteran’s Drive, University of Kentucky, Lexington, Kentucky 40546 USA

**Keywords:** Gene rearrangement, mitochondria, tRNA, homoplasy, convergence, phylogeny

## Abstract

Helicoidea is a diverse group of land snails with a global distribution. While much is known regarding the relationships of helicoid taxa, comparatively little is known about the evolution of the mitochondrial genome in the superfamily. We sequenced two complete mitochondrial genomes from *Praticolella mexicana* Perez, 2011 representing the first such data from the helicoid family Polygyridae, and used them in an evolutionary analysis of mitogenomic gene order. We found the mitochondrial genome of *Praticolella mexicana* to be 14,008 bp in size, possessing the typical 37 metazoan genes. Multiple alternate stop codons are used, as are incomplete stop codons. Mitogenome size and nucleotide content is consistent with other helicoid species. Our analysis of gene order suggested that Helicoidea has undergone four mitochondrial rearrangements in the past. Two rearrangements were limited to tRNA genes only, and two involved protein coding genes.

## Introduction

Helicoidea (Mollusca, Gastropoda) is a globally distributed and diverse superfamily of terrestrial mollusks (reviewed in Razkin et al. 2015). It is part of the larger Stylommatophora, a clade that accounts for around 80% of all terrestrial mollusks (Klussmann-Kolb et al. 2008) and encompasses over 100 families. Helicoid snails possess a typical pulmonate body plan with two pairs of tentacles, a usually dextrally coiled shell, and a vascularized pallial cavity that functions as a lung (Beesley et al. 1998). Taxonomic and systematic classifications of Helicoidea have differed based on morphological, ecological, and molecular characters including select mitochondrial markers (Steinke et al. 2004, Manganelli et al. 2005, Groenenberg et al. 2011). Phylogenies based on entire mitochondrial genomes (Wang et al. 2014a, Deng et al. 2016) are limited in size and scope due to the paucity of helicoid data relative to other mollusk groups (e.g. Caenogastropoda).

The mitogenome serves as a powerful evolutionary tool given its small size and fast mutation rate relative to the nuclear genome (Avise et al. 1987, Saccone et al. 1999, Funk and Omland 2003). Mitogenome organization has several qualities that make it a valuable phylogenetic marker. For example, mitochondrial gene order and content can be highly variable in metazoans (Black and Roehrdanz 1998, Mindell et al. 1998, Weigert et al. 2016). Transposition of tRNA genes is more common than movement of protein coding or ribosomal RNA genes (Xu et al. 2006) and may be weighted accordingly during analysis (Bader et al. 2008). Variability in gene length, arrangement, and strand assignment can be examined as sets of phylogenetically informative characters (Gissi et al. 2008). Many mitochondrial gene order rearrangements also represent rare events that serve as homoplasy-free evidence of common ancestry (Boore and Brown 1998, Rokas and Holland 2000, Gribaldo and Philippe 2002) provided they carry sufficient phylogenetic information (Yang 1998). In mollusks, these classes of genetic variation can occur within the same family or genus (Milbury and Gaffney 2005, Rawlings et al. 2010). Helicoideans possess typical metazoan mitogenomes, with 37 genes organized among ribosomal (16S and 12S) and transfer RNA (22, including two each for leucine and serine) genes and 13 protein coding genes (Boore 1999). The degree of genetic rearrangements and variability within and among helicoid mitogenomes, however, is poorly understood.

**Figure 1.**
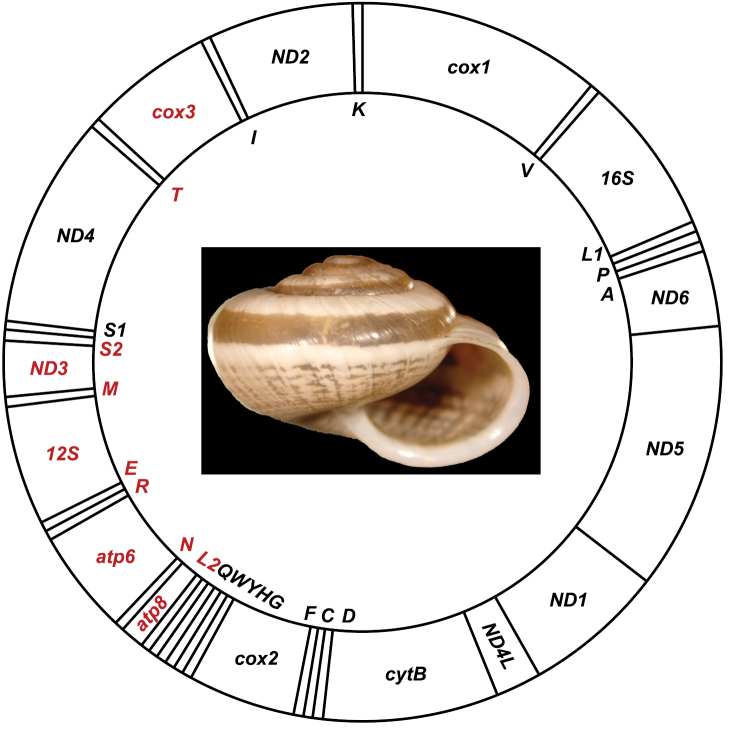
Mitochondrial genome of *Praticolella mexicana* UTRGV and McAllen illustrated with an image of the species holotype (ANSP 426031). Gene order and sizes are shown relative to one another, not including non-coding regions. Genes are color coded by H (black) or L (red) strand. IUPAC single letter codes are used to identify tRNA genes.

Within Helicoidea, only three of the constituent 19 families (Bouchet et al. 2005) are represented by complete mitogenomes on GenBank: Bradybaenidae, Camaenidae, and Helicidae. Currently unrepresented is Polygyridae, a helicoid family endemic to the Americas. Many of the most visible and commonly encountered land snails in North America are polygyrids. Nearly 300 species are described in the family, including five that are considered problematic invasives (Perez et al. 2014). We focused our efforts on *Praticolella mexicana* Perez, 2011, a small, globe-shaped snail (Figure 1) most likely native to Mexico and introduced to the Caribbean and the United States gulf coast (Perez 2011). The United States Department of Agriculture reports finding this species in shipments of fruit, furniture, and ornamental plants (USDA pers. comm.). Our study had two primary research aims: to sequence and annotate the mitochondrial genome of *Praticolella mexicana* to examine gene order and arrangement in Polygyridae; and to explore gene order evolution in Helicoidea. Results from both aspects of the study will increase our knowledge of these gastropod groups and provide a better understanding of land snail mitochondrial genome evolution.

## Materials and methods

### Specimen collection and DNA extraction

We collected one adult *Praticolella mexicana* each from the UTRGV campus in Edinburg, Texas (26.30726; -98.1714), and from a residential neighborhood in McAllen, Texas (26.2085; -98.2254). We immersed foot tissue from each snail in reconstituted BupH™ phosphate buffered saline (Thermo Scientific) and homogenized it with a Dounce homogenizer. We used EDTA-free Protease Inhibitor Cocktail (Thermo Scientific) and the Mitochondrial Isolation Kit for Tissue (Thermo Scientific) to isolate intact mitochondria from the homogenates. Mitochondrial DNA was extracted using the Mitochondrial DNA Isolation Kit (BioVision) followed by application of Plasmid-Safe™ ATP-dependent DNase (Epicenter) to remove any remaining nuclear DNA.

### Genome sequencing and assembly

We purified enriched mitochondrial DNA using the Zymoclean Genomic DNA Clean and Concentrator kit (Zymo Research) and quantified it using a BioAnalyzer (Agilent Technologies). Approximately 50 ng of total DNA was used for barcoded library construction using the Nextera DNA library prep kit (Illumina), precisely following the manufacturer’s instructions. We pooled the *Praticolella mexicana* samples on one flowcell and sequenced them on the MiSeq (Illumina) platform using the 2x250 bp run mode. Barcodes and deconvolution of the pooled reads was performed automatically in the BaseSpace (Illumina) server and used their native format. The CLCBio 8.0.2 *de novo* genome assembly tool was used to assemble the reads using default parameter settings. Genomic contigs representing mitochondrial DNA segments were subsequently identified using the CLCBio assembly Fasta files to query a BLAST database comprising the *Achatina fulica* mitochondrial genome (GenBank: KJ744205).

### Genome annotation

We loaded the assembly for each individual into Geneious 8 (http://www.geneious.com, Kearse et al. 2012) and used the built-in ORF finder function to identify putative coding regions. We compared the output to that generated in MITOS (Bernt et al. 2013) to determine the location and orientation of 13 protein-coding genes. ARWEN (Laslett and Canbäck 2008) and tRNAscan-SE 1.21 (Lowe and Eddy 1997) were used to identify the 22 tRNAs, and MITOS and BLAST (Altschul et al. 1990) were used to locate the two ribosomal genes. Nucleotide and codon composition analyses were conducted in DAMBE (Xia and Xie 2001).

### Phylogenetic analysis of mitochondrial genes

To determine the position of Polygyridae within Helicoidea, we extracted the amino acid sequences for all 13 protein-coding genes from the two new genomes. These were combined with mitochondrial genome sequences from eight other helicoid taxa, and eight stylommatophoran and one non-stylommatophoran outgroup (Table 1). Genome fragments of *Euhadra herklotsi* (Z71693-701) were excluded because the gene order data (see below) could not be coded. Data for each gene were aligned separately in MUSCLE (Edgar 2004). We used IQTREE 1.4.2 (Nguyen et al. 2014) to determine that all alignments fit the mtZOA+F+I+G4 model (Rota-Stabelli et al. 2009) optimally. We assembled all alignments into a single data matrix and analyzed it in IQTREE under maximum likelihood, allowing each gene to be optimized separately. We assessed branch support using 10,000 ultra-fast bootstrap replicates (Minh et al. 2013).

**Table 1.** Taxonomic list of mitochondrial genomes used in the study.

Taxonomy	GenBank	Reference
Clade Systellommatophora		
Superfamily Onchidioidea		
Family Onchidiidae		
*Onchidella celtica*	AY345048	Grande et al. 2004
Clade Stylommatophora		
Superfamily Achatinoidea		
Family Achatinidae		
*Achatina fulica*	KJ744205	He et al. 2016
Superfamily Clausilioidea		
Family Clausiliidae		
*Albinaria caerulea*	X83390	Hatzoglou et al. 1995
Superfamily Helicoidea		
Family Bradybaenidae		
*Aegista aubryana*	KT192071	Yang et al. 2016
*Aegista diversifamilia*	KR002567	Huang et al. 2016
*Dolicheulota formosensis*	KR338956	Huang et al. 2016
*Mastigeulota kiangsinensis*	KM083123	Deng et al. 2016
Family Camaenidae		
*Camaena cicatricosa*	KM365408	Wang et al. 2014a
Family Helicidae		
*Cepaea nemoralis*	U23045	Terrett et al. 1996
*Cylindrus obtusus*	JN107636	Groenenberg et al. 2012
*Cornu aspersum*	JQ417194	Gaitán-Espitia et al. 2013
Family Polygyridae		
*Praticolella mexicana* McAllen	KX259343	this study
*Praticolella mexicana* UTRGV	KX278421	this study
Superfamily Orthalicoidea		
Family Cerionidae		
*Cerion incanum*	KM365085	unpublished
Family Orthalicidae		
*Naesiotus nux*	KT821554	Hunter et al. 2016
Superfamily Pupilloidea		
Family Pupillidae		
*Gastrocopta cristata*	KC185403	Marquardt 2013
*Pupilla muscorum*	KC185404	Marquardt 2013
Family Vertiginidae		
*Vertigo pusilla*	KC185405	Marquardt 2013
Superfamily Succineoidea		
Family Succineidae		
*Succinea putris*	JN627206	White et al. 2011

### Gene order analysis and phylogeny

For all included mitogenomes, we determined the gene order and strand assignment. Using *Cornu aspersum* (JQ417194) as reference (Gaitán-Espitia et al. 2013), we numbered the genes consecutively in a 5’ to 3’ direction on the H strand starting with *cox1* as gene number one. With each of the genes now numbered, we proceeded to generate gene order for the other mitogenomes, using positive numbers for H strand and negative numbers for L strand. The resulting data matrix was analyzed under maximum likelihood using MLGO (Hu et al. 2014). MLGO uses a binary encoding method with probabilistic models (Lin et al. 2013) to infer gene duplications, genome rearrangements, and branch support through bootstrapping. We compared our amino acid phylogeny to the gene order tree using the Kishino-Hasegawa test (Kishino and Hasegawa 1989) as implemented in IQTREE. We also used our protein sequence phylogeny with MLGO to reconstruct ancestral genomes to study the evolution of gene order in Helicoidea.

## Results

Approximately 12 million sequences reads derived from the UTRGV *Praticolella mexicana* sample assembled into over 450,000 contigs, the largest of which was 14,275 bp in length. A BLAST search against the *Achatina fulica* mitogenome revealed that the largest contig was comprised entirely of mitochondrial sequence. The McAllen *Praticolella mexicana* sample comprised 26 million reads that assembled into more than 300,000 contigs. The largest contig spanned 14,259 bp and was also composed entirely of mitochondrial sequence. After final sequence editing, both *Praticolella mexicana* mitogenomes were found to be 14,008 bp in length.

The complete *Praticolella mexicana* mitochondrial genomes (KX278421 UTRGV, KX259343 McAllen; Figure 1) possess the same genes in the same orders and orientations (Table 2). The smallest tRNA is 54 bp (*tRNA-Ser1*), while the largest is 68 bp (*tRNA-Ser2*). Non-coding regions make up 1.4% (194 bp) of the *Praticolella mexicana* mitogenome. The genome has three large non-coding regions. The first region is 25 bp and sits between *cox3* and *tRNA-Ile*. The second region is 56 bp long and exists between the two serine tRNAs. The third region is a GC-rich 89 bp segment between the *tRNA-Trp* and *tRNA-Gln* genes. Searches using BLAST (Altschul et al. 1990) found no significant matches in any database for these three non-coding regions.

**Table 2.** Mitochondrial genome annotation for *Praticolella mexicana* UTRGV.

Gene	Start	Stop	Length	Strand	Start codon	Stop codon	Anticodon
*cox1*	1	1525	1525	H	TTG	T	
*tRNA-Val*	1526	1587	62	H			TAC
*16S*	1589	2579	991	H			
*tRNA-Leu1*	2580	2642	63	H			TAG
*tRNA-Pro*	2640	2706	67	H			TGG
*tRNA-Ala*	2706	2768	63	H			TGC
*ND6*	2769	3239	471	H	GTG	TAA	
*ND5*	3223	4893	1671	H	TTG	TAG	
*ND1*	4887	5768	882	H	ATC	TAG	
*ND4L*	5768	6052	285	H	GTG	TAA	
*cytB*	6054	7148	1095	H	ATT	TAG	
*tRNA-Asp*	7149	7212	64	H			GTC
*tRNA-Cys*	7209	7265	57	H			GCA
*tRNA-Phe*	7270	7331	62	H			GAA
*cox2*	7332	8003	672	H	ATG	TAG	
*tRNA-Gly*	8007	8068	61	H			TCC
*tRNA-His*	8063	8123	61	H			GTG
*tRNA-Tyr*	8131	8192	62	H			GTA
*tRNA-Trp*	8186	8248	63	H			TCA
*tRNA-Gln*	8338	8396	59	L			TTG
*tRNA-Leu2*	8397	8454	58	L			TAA
*atp8*	8458	8608	151	L	ATG	T	
*tRNA-Asn*	8612	8670	59	L			GTT
*atp6*	8671	9322	652	L	ATG	T	
*tRNA-Arg*	9323	9382	60	L			TCG
*tRNA-Glu*	9383	9442	60	L			TTC
*12S*	9443	10186	744	L			
*tRNA-Met*	10187	10248	62	L			CAT
*ND3*	10250	10597	348	L	TTG	TAA	
*tRNA-Ser2*	10598	10665	68	L			TGA
*tRNA-Ser1*	10723	10776	54	H			GCT
*ND4*	10777	12100	1324	H	ATG	T	
*tRNA-Thr*	12010	12162	62	L			TGT
*cox3*	12163	12958	796	L	ATT	T	
*tRNA-Ile*	12985	13044	60	H			GAT
*ND2*	13048	13954	907	H	ATG	T	
*tRNA-Lys*	13955	14008	61	H			TTT

Genome size for *Praticolella mexicana* is comparable with the other helicoid and stylommatophoran taxa available (Table 3). The majority of the genes (nine protein-coding, one rRNA, 15 tRNA) are located on the H strand (Figure 1). Gene overlap exists among protein coding and tRNA genes. The overall base composition of all taxa based on the H strand shows anti-cytosine bias along with excesses of thymine and guanine (Table 3). Protein-coding genes comprise 76.2% of the total *Praticolella mexicana* genome, and five start (ATC, ATG, ATT, GTG, TTG) and two stop (TAA, TAG) codons are used. Incomplete stop codons (T) are used for *atp6*, *atp8*, *cox1*, *cox3*, *ND2*, and *ND4* (Table 2). No downstream stop codons were found for those six genes.

**Table 3.** Nucleotide and skew statistics for the mitochondrial genomes used.

		Whole genome composition						
Species	Size (bp)	A%	C%	G%	T%	A+T%	AT skew	GC skew
*Achatina fulica*	15057	0,280	0,171	0,195	0,355	0,634	-0,118	0,064
*Aegista aubryana*	14238	0,313	0,145	0,164	0,379	0,692	-0,095	0,062
*Aegista diversifamilia*	14039	0,325	0,133	0,157	0,386	0,711	-0,086	0,083
*Albinaria caerulea*	14130	0,328	0,138	0,155	0,379	0,707	-0,073	0,059
*Camaena cicatricosa*	13843	0,319	0,135	0,167	0,379	0,698	-0,086	0,108
*Cepaea nemoralis*	14100	0,262	0,189	0,213	0,336	0,598	-0,125	0,058
*Cerion incanum*	15177	0,298	0,158	0,185	0,360	0,657	-0,095	0,077
*Cylindrus obtusus*	14610	0,258	0,166	0,219	0,358	0,615	-0,162	0,137
*Dolicheulota formosensis*	14237	0,284	0,131	0,167	0,418	0,702	-0,191	0,120
*Gastrocopta cristata*	14060	0,308	0,136	0,172	0,384	0,692	-0,110	0,116
*Helix aspersa*	14050	0,307	0,136	0,165	0,392	0,699	-0,121	0,097
*Mastigeulota kiangsinensis*	14029	0,295	0,144	0,182	0,379	0,674	-0,125	0,118
*Naesiotus nux*	15197	0,336	0,120	0,147	0,397	0,733	-0,083	0,100
*Praticolella mexicana* McAllen	14008	0,289	0,126	0,188	0,398	0,686	-0,159	0,198
*Praticolella mexicana* UTRGV	14008	0,288	0,126	0,188	0,398	0,686	-0,160	0,198
*Pupilla muscorum*	14149	0,325	0,129	0,153	0,393	0,718	-0,094	0,083
*Succinea putris*	14092	0,339	0,109	0,122	0,430	0,769	-0,113	0,055
*Vertigo pusilla*	14078	0,326	0,123	0,155	0,397	0,722	-0,098	0,116

**Figure 2.**
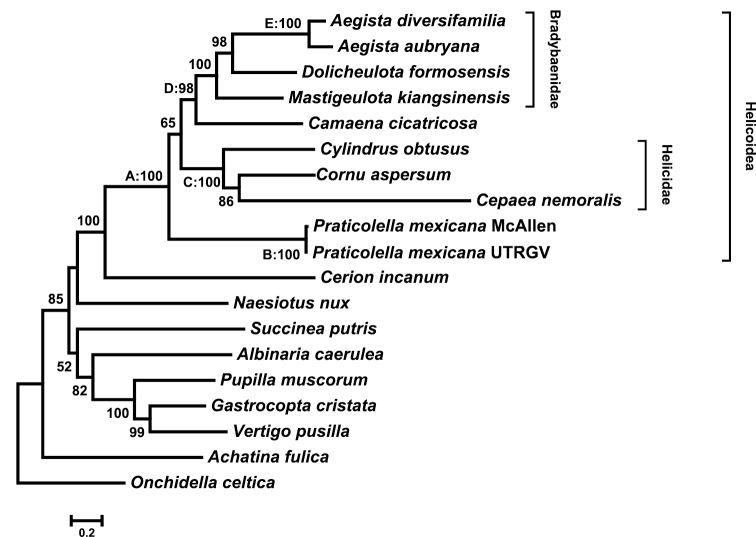
Maximum likelihood phylogeny of Stylommatophora protein coding genes. Analysis in IQTREE yielded a single tree (log likelihood = -89104.188) under the mtZOA+F+I+G4 model. Branch support >50% is shown based on 10,000 ultra-fast bootstrap replicates. Helicoidea, Bradybaenidae, and Helicidae were recovered as monophyletic. Nodes A-E refer to rearrangements shown in Figure 4.

**Figure 3.**
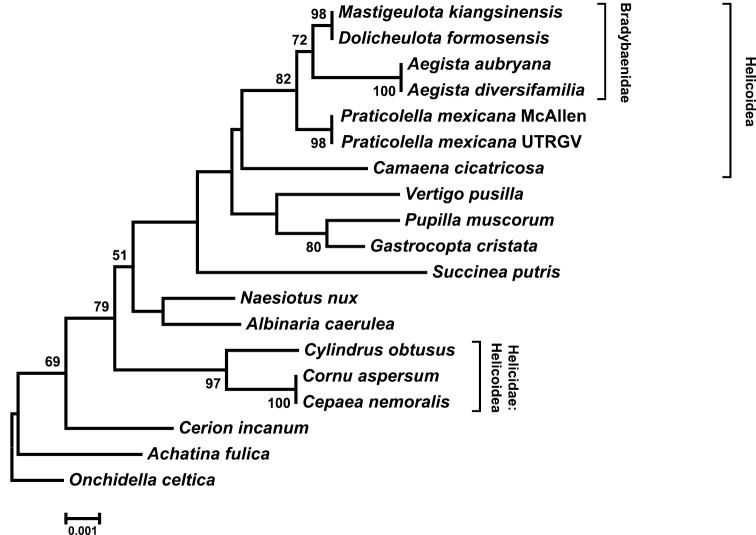
Maximum likelihood phylogeny of gene order. Analysis in MLGO yielded a single tree. Branch support >50% is shown based on 100 bootstrap replicates. Bradybaenidae and Helicidae were recovered as monophyletic, but Helicoidea was not.

Maximum-likelihood analysis of our protein sequence dataset of 19 taxa and 4,011 aligned amino acid positions yielded a single tree (Figure 2). Helicoidea, Bradybaenidae, and Helicidae were recovered as well-supported monophyletic groups (100% bootstrap support), as were Pupilloidea and Pupillidae. Bradybaenidae and *Camaena* were well-supported sister taxa, and *Praticolella mexicana* was positioned as sister to the remaining helicoid taxa. A maximum likelihood phylogeny of gene order again supported the monophyly of Bradybaenidae, Helicidae and Pupillidae, and suggested a sister relationship of Bradybaenidae with *Praticolella mexicana* (Figure 3). Helicoidea, well-supported in the analysis of protein sequences, was not recovered as monophyletic though branch support was low (Figure 2). The gene order phylogeny represented a significantly less likely topology than the protein phylogeny (Δ likelihood = 1246.275, *p* << 0.01).

**Figure 4.**
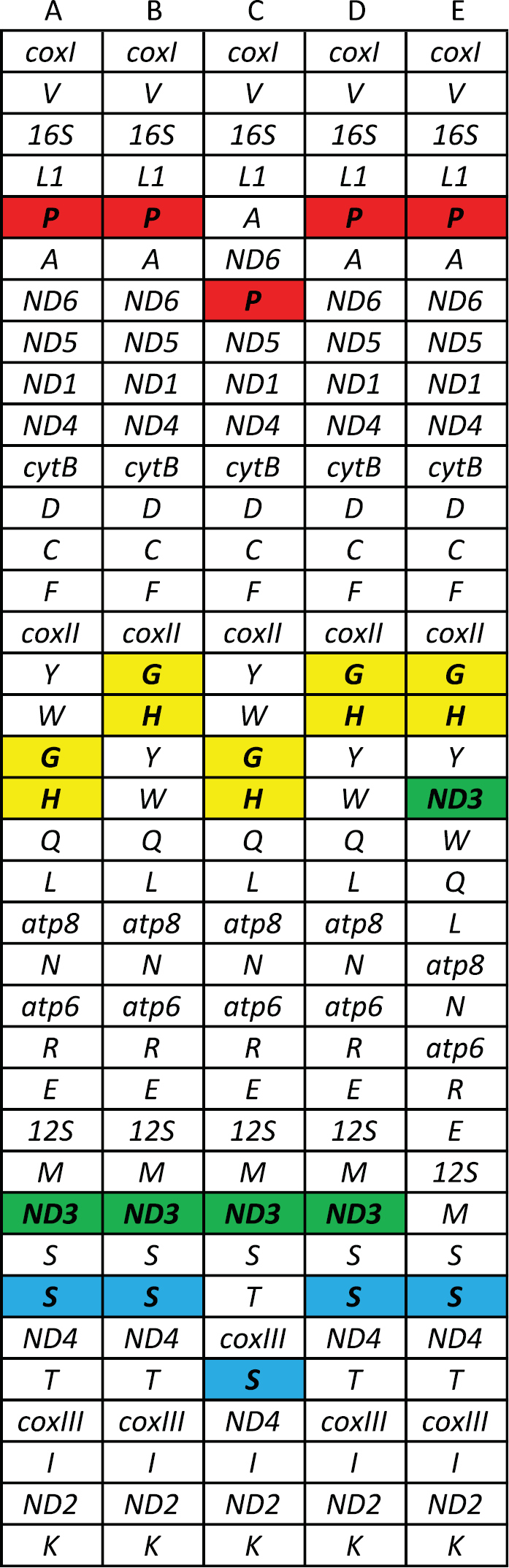
Ancestral gene order reconstructions for Helicoidea. Columns (**A–E**) correspond to labeled nodes in Figure 2. IUPAC single letter codes are used to identify tRNA genes. Rearrangements in red and blue are unique to Helicidae. The convergent rearrangement seen in Bradybaenidae, *Camaena*, and *Praticolella* is shown in yellow. The green rearrangement is unique to *Aegista*.

Given the protein topology represented the more likely relationships among our included taxa, we reconstructed ancestral gene orders predicted by MLGO with that topology. The results suggested a pattern of four rearrangements in the helicoid mitochondrial genome (Figure 4), assuming the fewest number of gene rearrangements. Starting with the hypothetical ancestral helicoid mitogenome (Figure 2 node A), *Praticolella mexicana* had (*tRNA-Tyr*, *tRNA-Trp*) transposing with (*tRNA-Gly*, *tRNA-His*). The Helicidae+*Camaena*+Bradybaenidae ancestor maintained the same order as the hypothetical ancestral helicoid. Two unique rearrangements followed in Helicidae (Figure 2 node C); *tRNA-Pro* moved from between *tRNA-Leu1* and *tRNA-Ala* to between *ND6* and *ND5*, and (*tRNA-Ser2*, *ND4*) transposed with *(tRNA-Thr, coxIII*). The ancestor of *Camaena*+Bradybaenidae (Figure 2 node D) showed the same (*tRNA-Tyr*, *tRNA-Trp*) and (*tRNA-Gly*, *tRNA-His*) rearrangement as *Praticolella mexicana*. Finally, a unique rearrangement is seen in *Aegista* (Figure 2 node E) The *ND3* gene moved from between *tRNA-Met* and *tRNA-Ser1* to between *tRNA-Tyr* and *tRNA-Trp*.

## Discussion

Gastropod mitogenomes tend to be compact (Boore 1999) even while carrying non-coding regions of varying sizes (Grande et al. 2008). Both mitogenomes sequenced from *Praticolella mexicana* encode the standard 37 metazoan genes and possess intergenic non-coding regions. We believe that the 56 bp non-coding region between *tRNA-Ser1* and *Ser2* may represent the putative mitochondrial origin of replication (POR) and control region for *Praticolella mexicana*. The POR is usually an AT-rich sequence that may contain palindromic stretches of nucleotides. The 56 bp region in *Praticolella mexicana* is comparable in size to the presumed POR in *Camaena* and Bradybaenidae and is similarly AT-rich, though it is often adjacent to *cox3* in pulmonate snails (Gaitán-Espitia et al. 2013). Both new mitogenomes have the same gene order and strand orientations, and have the highest GC skew and third highest AT skew (Perna and Kocher 1995) among the species examined. Strand-specific bias in nucleotide composition is a common feature among metazoan mitogenomes (Hassanin et al. 2005) and may be a constraint of organellar function (Asakawa et al. 1991).

The two *Praticolella mexicana* mitogenomes differ by 71 bp, which was fewer differences than seen in *Cornu aspersum* from Chile (107-149 bp), the only other stylommatophoran with more than one mitogenome available (Gaitán-Espitia et al. 2013). Our preliminary intraspecific mitogenome divergence in *Praticolella mexicana* (0.5%) is comparable to that seen in other metazoans such as butterfly (Vanlalruati et al. 2015) and catfish (Wang et al. 2014b) species. The majority of the differences represent substitutions in non-coding regions or third codon positions. Our results also show that *Praticolella mexicana* uses six different start codons and incomplete stop codons for mitochondrial gene expression. The invertebrate mitochondrial genetic code uses multiple alternate start codons (Osawa et al. 1992, Jukes and Osawa 1993), and partial stop codons are found in other land snail mitogenomes (Groenenberg et al. 2012, Wang et al. 2014a, Yang et al. 2015). Post-transcriptional adenylation is predicted to complete incomplete stop codons (Ojala et al. 1981).

Mitochondrial gene rearrangements are common across Metazoa (Black and Roehrdanz 1998, Boore et al. 2004, Mwinyi et al. 2009, Wang et al. 2016) often including the movement of tRNA genes (Mueller and Boore 2005, Dowton et al. 2009). In Helicoidea, two of the four rearrangements we observed involve tRNA genes only. These tRNA rearrangements are the most common type seen in mitogenomes (Xu et al. 2006) The (*tRNA-Tyr*, *tRNA-Trp*) and (*tRNA-Gly*, *tRNA-His*) rearrangement seen in bradybaenids, camaenids, and polygyrids represents a convergent restructuring of the genome. These homoplastic events involving tRNA genes were first observed in insects (Flook et al. 1995) and have been reported across Arthropoda. The fourth rearrangement we observed is unique to *Aegista*, involving the movement of the *ND3* protein coding gene. While less frequent than tRNA rearrangements, those involving protein coding genes without multiple gene inversions or transpositions have been shown in other mollusks (Grande et al. 2008, Osca et al. 2015).

Our protein sequence data support previous works showing the monophyly of Helicoidea, Helicidae, and Bradybaenidae. Previous work has suggested close relationships between Bradybaenidae, Camaenidae, and Polygyridae, but the monophyly of the former two families remains in question (Wade et al. 2001, Cuezzo 2003, Wade et al. 2006, Wade et al. 2007, Razkin et al. 2015). Unfortunately, with the relatively small number of mitogenomes available, our data were unable to resolve these issues. Our gene order phylogeny further supported the monophyly of Helicidae and Bradybaenidae, suggested close relationships between Bradybaenidae, *Camaena*, and *Praticolella mexicana*, but did not suggest a monophyletic Helicoidea despite its consistent recovery elsewhere (Wade et al. 2001, Grande et al. 2004, Wade et al. 2007, Razkin et al. 2015). This finding was unexpected, since comparisons of gene trees to gene order phylogenies tend to produce similar results across the tree of life when using whole nuclear genomes (Wolf et al. 2001). Differences that do arise have been attributed to the phylogenies deriving from uncorrelated datasets, with gene sequence trees being driven by point mutations in the coding DNA sequence while the gene orders vary through rearrangement (Sankoff et al. 1992). While many studies examine mitochondrial gene order in the context of sequence phylogenies (e.g. Aguileta et al. 2014, Weigert et al. 2016), we found no studies that used MLGO or similar older programs (e.g. MGRA, Alekseyev and Pevzner 2009) to generate mitochondrial gene order phylogenies. More thorough and inclusive analyses are needed to determine to what extent the two types of phylogenies can be congruent and how that congruence speaks to mitogenomic evolution (Leigh et al. 2011).
